# Appropriate Usage of Face Masks to Prevent SARS-CoV-2: Sharpening the Messaging Amid the COVID-19 Pandemic

**DOI:** 10.1017/dmp.2020.336

**Published:** 2020-09-10

**Authors:** Karina Escandón, Graham P. Martin, Krutika Kuppalli, Kevin Escandón

**Affiliations:** Department of Anthropology, Universidad Nacional de Colombia, Bogotá, Colombia; The Healthcare Improvement Studies Institute (THIS Institute), University of Cambridge, Cambridge, United Kingdom; Division of Infectious Diseases, Medical University of South Carolina, Charleston, South Carolina; School of Medicine, Universidad del Valle, Cali, Colombia

**Keywords:** coronavirus, COVID-19, mask, public health, SARS-CoV-2

**FIGURE 1:**
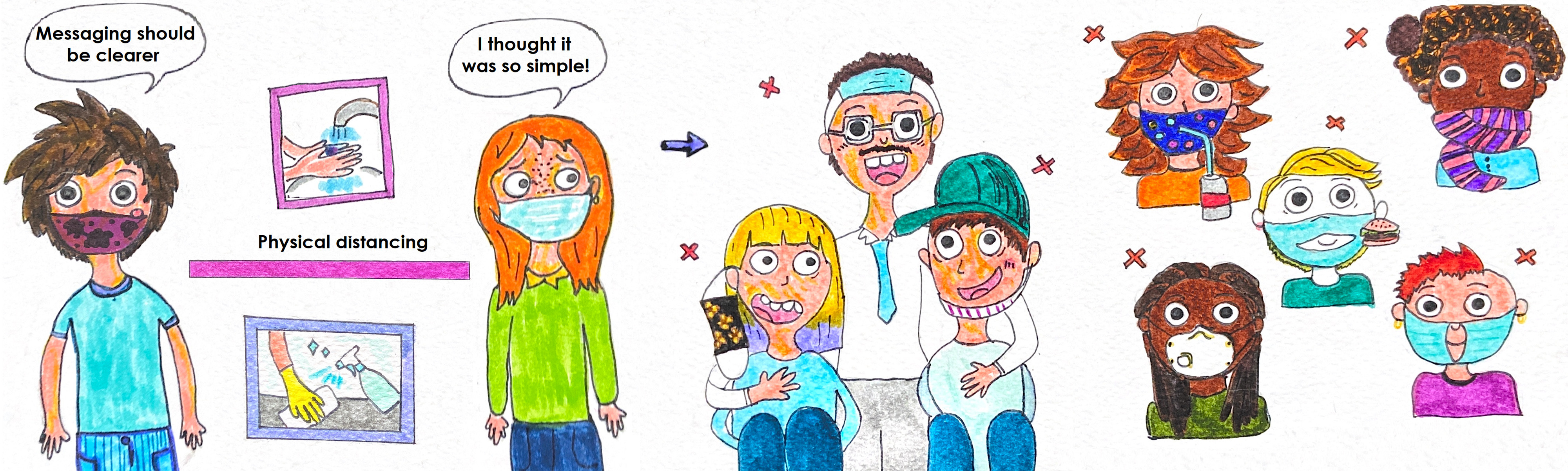
Appropriate and Inappropriate Mask Wearing Amid the COVID-19 Pandemic.

Many governments currently recommend or mandate universal use of face masks amid the coronavirus disease 2019 pandemic. Cloth face masks and makeshift face coverings – from bandanas and scarves to do-it-yourself and commercially available masks – are being advised for source control in many countries and regions to mitigate severe acute respiratory syndrome coronavirus 2 (SARS-CoV-2) transmission. Unfortunately, instances of individuals inappropriately wearing masks are being witnessed in public and on social media as they have become part of our “new normal” (Figure 1).

The directives from some public health officers and the messaging by some mask advocates, though well-intended, have been overly simplistic with slogans, such as “Wear a face covering, something is better than nothing,” “Just wear a mask, it’s common sense,” and “The science behind masks is simple and clear.”^1-3^ Also, polarizing narratives and the broad use of terms such as *face covering* have led to misconceptions and conflicting messages about the types of masks and the instructions for their use.

The use of masks is not intuitive, and the science behind them is not straightforward.^4,5^ Whereas simplistic and overconfident messaging is misleading, accurate messaging about the benefits, risks, and uncertainties is essential to gaining public support for the uptake of non-pharmaceutical interventions and thus the COVID-19 response. Based on available filtration efficacy data, the degree of protection from cloth masks is variable and greatly hinges on mask composition, thickness, permeability, electrostatic properties, and facial fit.^5-7^ Furthermore, masks require careful instructions (Table 1) to improve the likelihood of achieving their real-world benefits and to avoid the potential for self-contamination and mask contamination with viral particles.^6,8-10^ Inappropriate mask wearing is likely to reduce the effectiveness of masks. Several studies have reported the challenges related to appropriate mask use, compliance, and acceptance.^9^ Mask adherence does not simply reflect discipline; knowledge, risk perception, social acceptability, values, preferences, trust, and accessibility and affordability of masks play a major role.

**TABLE 1:** Instructions for Optimal Mask Wearing, Storage, and Disposal^6,8-10^

Wash hands thoroughly with water and soap for at least 20 seconds or clean them using an alcohol-based hand sanitizer (only if hands are not visibly dirty) before wearing a mask and after touching or removing the mask. Put the mask on and ensure that it fits snugly around the face and covers the nose, mouth, and chin completely at all times while wearing it. Avoid touching the face and the mask. If you touch the mask, wash or sanitize your hands. Do not fiddle with the mask. Do not share a mask with others. Remove the mask from the tie straps behind the head or the elastic ear loops, without grabbing the front. Discard single-use masks immediately upon removal or when it becomes damaged, moist (eg, contaminated with bodily fluids and secretions), or visibly dirty. Routinely wash reusable cloth masks with regular detergent and let them dry before wearing again. Keep masks in a clean, dry place (eg, a clean plastic or paper bag). Since moisture from breathing inside masks may affect their effectiveness, it should be cautioned against the use of a single mask for extended periods. It is recommended to change to a fresh mask when the mask being worn becomes damp. Masks should not be worn by children under age 2 years, people who may have baseline breathing difficulties, and people who are unconscious, incapacitated, or otherwise unable to wear a mask correctly. Alternatively, these people should rely on keeping the respiratory etiquette along with all other personal and environmental hygienic measures. Primary school-aged children should have adult supervision when wearing a mask. Masks should not be seen as an alternative to physical distancing.

Health education, effective scientific communication, context-sensitive policymaking, and community engagement are key tools to enhancing the understanding of masks’ benefits and downsides, encouraging health-seeking behaviors, warning against complacency, building trust in public health agencies, and avoiding judgment and shaming. Diverse and tailored information campaigns (eg, mainstream press, social media, public advertising, training seminars) and community outreach programs that incorporate surveillance around mask adherence practices are needed. Likewise, potential unintended consequences should be studied, anticipated, and addressed to maximize any benefits and minimize any downsides of widespread mask use.^9^ Amid this pandemic, there are research opportunities on different approaches to information provision; attitudes, behaviors, and beliefs toward proper mask wearing; and cloth mask designs, duration of use, reuse, breathability, and disinfection methods.^4^

In this article, we challenge inaccurate communication on community masking and argue for the need to improve public health messaging. In contrast to the abovementioned statements, more accurate statements are: “Wearing a cloth mask correctly may help slow down viral spread,” “Wear a cloth mask when in enclosed or crowded spaces,” and “Help reserve medical masks for health care workers by not hoarding them.” Such messages provide clarity while acknowledging nuances and lingering uncertainties, rather than providing oversimplified mask indications or misleading accounts regarding the effectiveness of face masks.
